# Clinical and Transfusion Approach to Neonatal Thrombocytopenia Associated With Systemic Lupus Erythematosus: A Case Report

**DOI:** 10.7759/cureus.88979

**Published:** 2025-07-29

**Authors:** Alejandra G Rosado-Ordaz, Paulina Espadas-Sauri, Victor M Ayuso-Diaz

**Affiliations:** 1 Faculty of Medicine, Universidad Autónoma De Yucatán (UADY), Mérida, MEX; 2 Department of Pediatrics, Institute for Social Security and Services for State Workers (ISSSTE) - Hospital Regional "Elvia Carrillo Puerto", Mérida, MEX; 3 Research and Education Division, Medical Care and Research, Mérida, MEX; 4 Genomic-Metabolic Unit, Marista University of Mérida, Mérida, MEX

**Keywords:** antinuclear antibodies, autoimmune cytopenia, immune thrombocytopenic purpura, maternal-fetal interface, neonatal intensive care, neonatal lupus, neonatal thrombocytopenia, postpartum immunology, systemic lupus erythematosus

## Abstract

Autoimmune diseases present a clinical challenge for young women of childbearing age since pregnancy can affect their progression and lead to complications for both mother and baby. Systemic lupus erythematosus (SLE) is of particular interest in this context due to its association with an increased risk of spontaneous abortion, foetal death, pre-eclampsia, intrauterine growth restriction, preterm delivery, and various neonatal manifestations, including thrombocytopenia. In this context, autoimmune neonatal thrombocytopenia, caused by the transplacental transfer of maternal autoantibodies, may be the first sign of an undiagnosed autoimmune disease in the mother. This report documents the clinical and transfusion management of severe neonatal thrombocytopenia in a pregnancy involving a mother with a history of idiopathic thrombocytopenic purpura. Subsequent study revealed positive serology for SLE. The favourable outcome for the neonate following intensive management underscores the importance of promptly identifying this condition and adopting a multidisciplinary approach to minimise the risk of severe haemorrhagic complications.

## Introduction

Autoimmune diseases predominantly affect women of childbearing age, and pregnancy poses clinical challenges due to the immunological and hormonal changes that can exacerbate disease activity, affecting both the mother and the foetus [1-5]. Patients with systemic lupus erythematosus (SLE) are at increased risk of maternal-fetal complications such as recurrent miscarriage, intrauterine growth restriction, preeclampsia, and preterm birth, which can lead to neonatal complications including low birth weight, respiratory distress, and thrombocytopenia [3,6-8].

During gestation, the bidirectional exchange of maternal and fetal cells, known as maternal-fetal microchimerism, can trigger immune responses in the fetus or induce autoimmune processes in the neonate. This phenomenon is particularly relevant in autoimmune neonatal thrombocytopenia, where the transplacental transfer of maternal autoantibodies against foetal platelet glycoproteins causes their accelerated destruction [1,3,5].

Neonatal thrombocytopenia is defined as a platelet count of less than 150,000/mm³ and affects 1-5% of healthy newborns. Its prevalence can reach 35% in neonates admitted to intensive care units (ICUs) [1-3]. Prematurity is a major risk factor, with thrombocytopenia affecting 70-80% of preterm low birth weight neonates [2]. Platelet counts tend to be lower in the first hours of life, reaching minimum values between two and five days, before progressively normalising around the seventh day. In some cases, physiological thrombocytopenia can be confused with pathological forms, such as the so-called 'neonatal fictitious thrombocytopenia,' which is observed in 29% of neonates weighing less than 1500 g [4-6]. However, up to 25% of cases may progress to severe forms (count <50,000/mm³), which increases the risk of intracranial haemorrhage, the most feared complication in these patients [4-6].

The etiological study of neonatal thrombocytopenia is essential to differentiate between immune and non-immune causes. In term neonates, immune causes are the most common and can be classified into alloimmune thrombocytopenia, secondary to maternal-fetal incompatibility of platelet antigens (e.g., HPA-1a), and autoimmune thrombocytopenia, caused by transplacental transfer of maternal autoantibodies, as occurs in idiopathic thrombocytopenic purpura (ITP) and SLE [9-14].

The treatment of neonatal thrombocytopenia depends on the severity of the condition. In cases of severe thrombocytopenia (<50,000/mm³) or with active bleeding, platelet transfusion is the treatment of choice, with recommended doses of 10-20 ml/kg [7,8]. Other strategies include administration of intravenous immunoglobulin G (1 g/kg/day) and corticosteroids, mainly methylprednisolone, which have shown efficacy in modulating the immune response and preventing hemorrhagic complications [9-11].

Early detection and awareness of neonatal autoimmune thrombocytopenia are critical because many mothers are asymptomatic, which can delay diagnosis and timely intervention. Identifying neonates at increased risk of severe thrombocytopenia early on and managing them appropriately can significantly improve clinical outcomes [12,13,15].

## Case presentation

We present the case of a 30-year-old female patient in her second pregnancy, who was diagnosed with chronic ITP at the age of 13 years. She was treated with systemic steroids for eight years, during which time she did not require a splenectomy or experience a relapse. Her first pregnancy was uneventful. 

In the current pregnancy, she presented with a urinary tract infection at 18 weeks, which was treated as an outpatient. At 25 weeks, she was hospitalised due to thrombocytopenia, with a platelet count of 48,000/μL. At that time, ITP was suspected, as she had no other autoimmune symptoms and no immunological workup had previously been conducted. She received intravenous methylprednisolone and was discharged on oral prednisone (25 mg/day).

At 35 weeks, she was admitted to the obstetric emergency department due to the spontaneous onset of labour, characterised by the expulsion of a bloody mucus plug and regular uterine contractions. Physical examination revealed cervical dilatation of 3 cm, intact membranes, and preserved uterine dynamics.

A complete blood count (CBC) performed on the mother's admission revealed severe thrombocytopenia, with a platelet count of 3,600/μL (Table 1). Due to the high risk of maternal-fetal haemorrhage, it was decided to terminate the pregnancy via caesarean section. Prior to surgery, the patient received 90 g of intravenous immunoglobulin G and 20 units of platelet concentrate. Despite this intervention, however, the platelet count remained critically low (below 20,000/μL) prior to surgery.

**Table 1:** Maternal blood biometry on admission

Parameter	Result	Reference Range
Hemoglobin (g/dL)	11.2	12.0 – 16.0
Hematocrit (%)	34	36 – 46
Platelets (/µL)	3,600	150,000 – 450,000
White Blood Cells (/µL)	9,800	4,500 – 11,000

A female neonate was delivered by caesarean section at 36 weeks of gestation (Capurro), with APGAR scores of 8 and 9 at one and five minutes, respectively. The neonate weighed 2,730 g, measured 45 cm in length, and had a head circumference of 33 cm. No external malformations were identified at birth, and she was initially managed conservatively.

Given the maternal history of severe thrombocytopenia and the high risk of neonatal immune cytopenias, the neonate was admitted to the neonatal intensive care unit (NICU) immediately after birth for close monitoring and evaluation. Eighteen hours later, she developed petechiae on the lower limbs and ecchymosis on the lumbar region. Blood biometry revealed severe thrombocytopenia (23,000/µL), while haemoglobin and haematocrit values remained within normal limits (Table 2). During the initial evaluation, mild abdominal distension was observed, and an orogastric tube was placed for drainage of blood-stained gastric contents. Within the first 24 hours of life, the baby also developed conjunctival haemorrhage and two episodes of apnoea, for which she received supportive management, including close monitoring and tactile stimulation. Due to the severity of thrombocytopenia and clinical bleeding, a platelet concentrate transfusion was administered. Transfontanellar ultrasound showed no evidence of intracranial haemorrhage.

**Table 2:** Neonatal blood biometry on admission to the NICU NICU: neonatal intensive care unit

Parameter	Result	Reference Range
Hemoglobin (g/dL)	14.8	14.0 – 24.0
Hematocrit (%)	44	42 – 65
Platelets (/µL)	23,000	150,000 – 450,000
White Blood Cells (/µL)	15,200	9,000 – 30,000

On the second day of extrauterine life, the neonate presented with sinus bradycardia (heart rate of 80 bpm), accompanied by poor peripheral perfusion, hypotonia, and mild desaturation (SpO₂ 88%), suggesting early haemodynamic compromise. A continuous dobutamine infusion was initiated to support cardiac output and improve tissue perfusion, and was discontinued after 24 hours once haemodynamic parameters stabilised.

Subsequently, she developed transient respiratory distress characterised by mild intercostal retractions and nasal flaring, attributed to fluid retention or delayed clearance of lung fluid, a common finding in late preterm neonates. However, she did not require supplemental oxygen or mechanical ventilation.

Due to clinical suspicion of early-onset neonatal sepsis, empirical antibiotic therapy with ampicillin and gentamicin was started after obtaining blood cultures. The neonate’s condition improved progressively, blood cultures remained sterile, and antibiotics were discontinued on day five. The neonate was discharged on day seven in stable condition and referred for outpatient paediatric follow-up.

During the puerperium, maternal immunological studies revealed the presence of antinuclear antibodies (ANA) and double-stranded anti-DNA, as well as hypocomplementemia. Therefore, the diagnosis of ITP was reclassified as SLE. This finding suggests an underlying autoimmune disease that was previously unidentified, whose activity may have intensified the fetal immune response and contributed to the severe neonatal thrombocytopenia.

**Figure 1:**
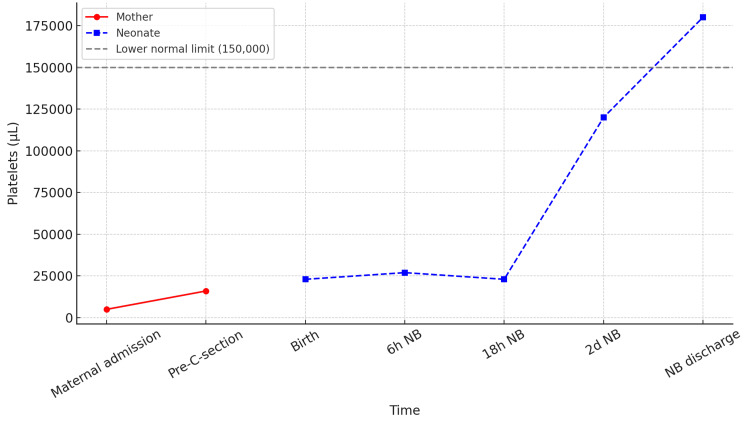
Evolution of the platelet count in the mother and the neonate during clinical and transfusion approaches. The red line represents the maternal platelet count at admission and prior to caesarean section (C-section). The blue line represents the neonatal platelet count at different time points: at birth, 6 hours after birth (6h NB), 18 hours after birth (18h NB), on the second day of life (2d NB), and at neonatal discharge (NB discharge). The dashed grey line marks the lower normal platelet limit (150,000/μL). NB: newborn; C-section: caesarean section; h: hours; d: days

Figure 1 shows the evolution of platelet counts in the mother and newborn throughout treatment, illustrating the response to therapeutic interventions including intravenous immunoglobulin and platelet transfusions, as well as the timing of delivery and subsequent haematological stabilisation of both patients. 

## Discussion

Immunological neonatal thrombocytopenia is an uncommon but highly relevant clinical condition, particularly in neonates born to mothers with autoimmune diseases such as SLE. It results from the transplacental passage of maternal autoantibodies, primarily IgG immunoglobulins that target fetal platelet antigens. This leads to the accelerated destruction of platelets in the neonate. In the presented case, the early onset (within hours after birth) of severe neonatal thrombocytopenia enabled the identification of an undiagnosed immunological condition in the mother, highlighting the diagnostic interdependence of the mother-child dyad in cases of perinatal immunopathology [16,17].

From a pathophysiological perspective, SLE is characterised by the sustained production of autoantibodies and the formation of circulating immunocomplexes, which is favoured by regulatory T-cell dysfunction and B-cell hyperactivity. During gestation, these autoantibodies can cross the placental barrier from the second trimester onwards. This can induce not only direct fetal tissue damage, as has been described in neonatal lupus, but also immune-mediated cytopenias. Thrombocytopenia is the most common of these [18,19].

Although neonatal lupus is classically associated with anti-Ro/La antibodies and may present with congenital atrioventricular block or cutaneous exanthema, in many cases, as in this one, the manifestations are restricted to the haematological system. Platelet counts below 30,000/µL, together with clinical signs of bleeding (e.g., petechiae, ecchymosis, and conjunctival haemorrhage), require an immediate approach due to the high risk of severe haemorrhagic complications such as intracranial haemorrhage [16,20].

The severity of neonatal thrombocytopenia observed in this patient (approximately 20,000/μL at birth, as shown in Figure 1), in conjunction with extreme maternal thrombocytopenia at delivery (3,600/μL), presents an unusual case. In women with SLE, platelet counts rarely drop below 50,000/μL in the absence of an acute flare [18]. However, the literature suggests that these isolated relapses may represent a haematological expression of subclinical immune activity, particularly in patients who have previously been diagnosed with ITP and subsequently meet serological criteria for SLE [3].

The case also demonstrates the usefulness of postpartum immunological screening. The presence of ANA and double-stranded anti-DNA, together with hypocomplementemia, meant that the maternal diagnosis could be reclassified as SLE. This change in diagnosis has therapeutic, reproductive, and prognostic implications, as SLE is a condition requiring long-term follow-up, particularly in women of childbearing age [17,18].

From a therapeutic point of view, both the mother and the newborn received interventions in accordance with current guidelines. In the mother, the administration of intravenous immunoglobulin (IVIG) and a platelet transfusion enabled the risk of peripartum haemorrhage to be controlled. In the neonate, immediate admission to the NICU, platelet transfusion, and timely haemodynamic support were crucial in avoiding serious adverse events. Notably, no neurological or cardiac manifestations were identified, and the clinical evolution was favourable without the need for ventilatory support. These interventions align with the algorithm proposed by Blanchette et al. for the management of neonatal alloimmune and autoimmune thrombocytopenia, which emphasises early transfusion support and IVIG therapy in neonates with platelet counts <30,000/μL or with bleeding manifestations [17,20].

In this case, a diagnostic limitation was the inability to detect specific antiplatelet antibodies in the neonatal blood, which would have permitted direct confirmation of the immunological mechanism. Nevertheless, the self-limiting clinical course, exclusion of infectious causes and pattern of progressive platelet count recovery support the diagnosis of neonatal autoimmune thrombocytopenia in the absence of direct serological confirmation, as documented in the literature [3].

Compared to other case series, the reverse pattern observed here, in which neonatal thrombocytopenia led to a definitive diagnosis in the mother, has been described previously, albeit sporadically. Gotesman et al. (2024) emphasise that neonatal immune cytopenias should be considered a 'diagnostic window' for silent maternal conditions such as lupus, autoimmune thyroiditis and antiphospholipid syndrome [17].

This case also highlights the importance of using evolving platelet count curves to monitor and evaluate therapeutic responses. Integrating these data into clinical registries improves decision-making and facilitates the early detection of relapses or complications. From a public health and preventive medicine perspective, postpartum immunological screening of mothers with an atypical haematological history could be incorporated as a complementary strategy in high-risk obstetrics units, particularly in regions where autoimmune diseases are prevalent and are often not diagnosed promptly.

## Conclusions

The simultaneous presentation of severe thrombocytopenia in both a pregnant woman and her newborn, in the absence of a confirmed maternal autoimmune disease diagnosis, constitutes a complex clinical scenario requiring rigorous aetiological analysis. This case exemplifies how a haematological manifestation in a newborn can reflect an unrecognised maternal immunological disorder, and how identifying it during the puerperium can alter the diagnostic and therapeutic approach for both patients. The favourable clinical response achieved through the use of intravenous immunoglobulin and intensive management reaffirms the importance of personalised therapeutic strategies in perinatal immunology settings.

## References

[1] Bayhan T, Tavil B, Korkmaz A (2016). Neonates born to mothers with immune thrombocytopenic purpura: a single-center experience of 20 years. Blood Coagul Fibrinolysis.

[2] Morrone K (2018). Thrombocytopenia in the newborn. NeoRev.

[3] Donato H (2021). Neonatal thrombocytopenia: a review. I. Definitions, differential diagnosis, causes, immune thrombocytopenia. Arch Argent Pediatr.

[4] Peng T, Shan Y, Zhang P, Cheng G (2022). Bleeding in neonates with severe thrombocytopenia: a retrospective cohort study. BMC Pediatr.

[5] Morillas Martínez N, Martínez Fernández E, Ferrer Arriazu M (2023). Neonatal immune thrombocytopenia, a little-known pathology and a diagnostic challenge [Article in Spanish]. Bol S Vasco-Nav Pediatr.

[6] Stanworth SJ, Mumford AD (2023). How I diagnose and treat neonatal thrombocytopenia. Blood.

[7] Álvarez M, Hurtado J (2019). Platelet transfusion in newborns. New evidence. New uncertainties [Article in Spanish]. Evid Pediatr.

[8] Resch E, Hinkas O, Urlesberger B, Resch B (2018). Neonatal thrombocytopenia-causes and outcomes following platelet transfusions. Eur J Pediatr.

[9] Provan D, Arnold DM, Bussel JB (2019). Updated international consensus report on the investigation and management of primary immune thrombocytopenia. Blood Adv.

[10] Alsaleem M (2020). Intravenous immune globulin uses in the fetus and neonate: a review. Antibodies (Basel).

[11] Batton E, Leibel SL (2022). Immune-mediated neonatal thrombocytopenia. Neoreviews.

[12] Matusiak K, Patriquin CJ, Deniz S (2022). Clinical and laboratory predictors of fetal and neonatal alloimmune thrombocytopenia. Transfusion.

[13] Provan D, Semple JW (2022). Recent advances in the mechanisms and treatment of immune thrombocytopenia. EBioMedicine.

[14] Norton T, Newberry D, Jnah A (2021). Neonatal alloimmune thrombocytopenia: a concise review. Adv Neonatal Care.

[15] Bundhun PK, Soogund MZ, Huang F (2017). Impact of systemic lupus erythematosus on maternal and fetal outcomes following pregnancy: a meta-analysis of studies published between years 2001-2016. J Autoimmun.

[16] Bussel JB, Knightly KA (2024). Immune thrombocytopenia (ITP) in pregnancy. Br J Haematol.

[17] Gotesman M, Shear M, Raheel S, Procassini M, Panosyan EH (2024). Pediatric immune thrombocytopenia. Adv Pediatr.

[18] Fanouriakis A, Tziolos N, Bertsias G, Boumpas DT (2021). Update οn the diagnosis and management of systemic lupus erythematosus. Ann Rheum Dis.

[19] Blanco S, Vega LC, Carrizo LH, Culasso JM, Gallego SV (2022). Fetal and neonatal alloimmune thrombocytopenia: a late or missed diagnosis disease in fetal and perinatal health-care settings. J Matern Fetal Neonatal Med.

[20] Zhou M, Sun W, Peng H, Zhu X (2024). Neonatal lupus erythematosus successfully treated by exchange transfusion: a case report and literature review. Front Pediatr.

